# Infection Control in Carceral Facilities

**DOI:** 10.1093/cid/ciaf479

**Published:** 2025-10-21

**Authors:** Amadin A Olotu, Joseph A Bick, B Sue Medley-Lane, Anne C Spaulding

**Affiliations:** Rollins School of Public Health, Emory University, Atlanta, Georgia, USA; California Department of Corrections and Rehabilitation, California Correctional Health Care Services, Davis, California, USA; Infection Prevention and Control, Centurion Health, Sterling, Virginia, USA; Rollins School of Public Health, Emory University, Atlanta, Georgia, USA; School of Medicine, Emory University, Atlanta, Georgia, USA

**Keywords:** infection prevention and control, prison, jail, carceral, correctional

## Abstract

At the end of 2022, 5.4 million people (1 of every 48 adults) were either in jail, in prison, or on probation/parole in the United States. One in 20 persons in the United States (5%) will be incarcerated during their lifetime. Among those newly admitted to carceral settings, the prevalence of active tuberculosis disease and infections from blood-borne viruses and sexually transmitted pathogens is substantially higher than in community counterparts. Exposure to the carceral setting places residents, employees, and visitors at risk for acquiring communicable diseases, predominately those transmitted via airborne routes. Efforts to prevent, mitigate, or control infectious diseases benefit those within carceral facilities and also surrounding communities. This review briefly explores some of the changes, challenges, and opportunities relevant to the prevention and control of infectious diseases in jails, prisons, and juvenile residential facilities. It also emphasizes the need for adequate education and training when planning and implementing interventions.

## Teaser

Carceral facilities face unique infection control issues due to higher infectious disease prevalence, overcrowding, and inadequate ventilation. Prevention and treatment benefit residents and surrounding communities. Key strategies include written policies, public health partnerships, and education of staff and the incarcerated.

**(See the Editorial Commentary by McDougal and Strick on pages e791–3.)**

In the almost 2 decades since a synopsis of infection control in jails and prisons was published in this journal [1], pertinent changes (Table 1) have occurred within the US carceral settings that impact infection prevention and control. Certain classic issues (Table 2) still confront infectious disease physicians and infection control practitioners serving in these settings or caring for patients transferred from a carceral institution (see Box 1 for definitions of terms associated with incarceration). Despite a decline in the numbers of those in custody [2], the United States continues to incarcerate a larger proportion of its population than most other nations [3]. Individuals entering or residing in carceral facilities are more likely to be infected with hepatitis C virus (HCV) [4], hepatitis B virus (HBV), human immunodeficiency virus (HIV), *Neisseria gonorrhoeae*, *Treponema pallidum*, *Chlamydia trachomatis*, and *Trichomonas vaginalis* [5], and have a higher incidence of tuberculosis (TB) [6–8] than the general population. The higher baseline prevalence of infections, overcrowding, poor ventilation, inadequate infection surveillance, insufficient resources, and lapses in enforcing infection control policies in many carceral facilities contribute to an increased risk of acquiring infections in these settings [1].

## POLICIES, EDUCATION, AND ENGAGEMENT

**Figure 1.**
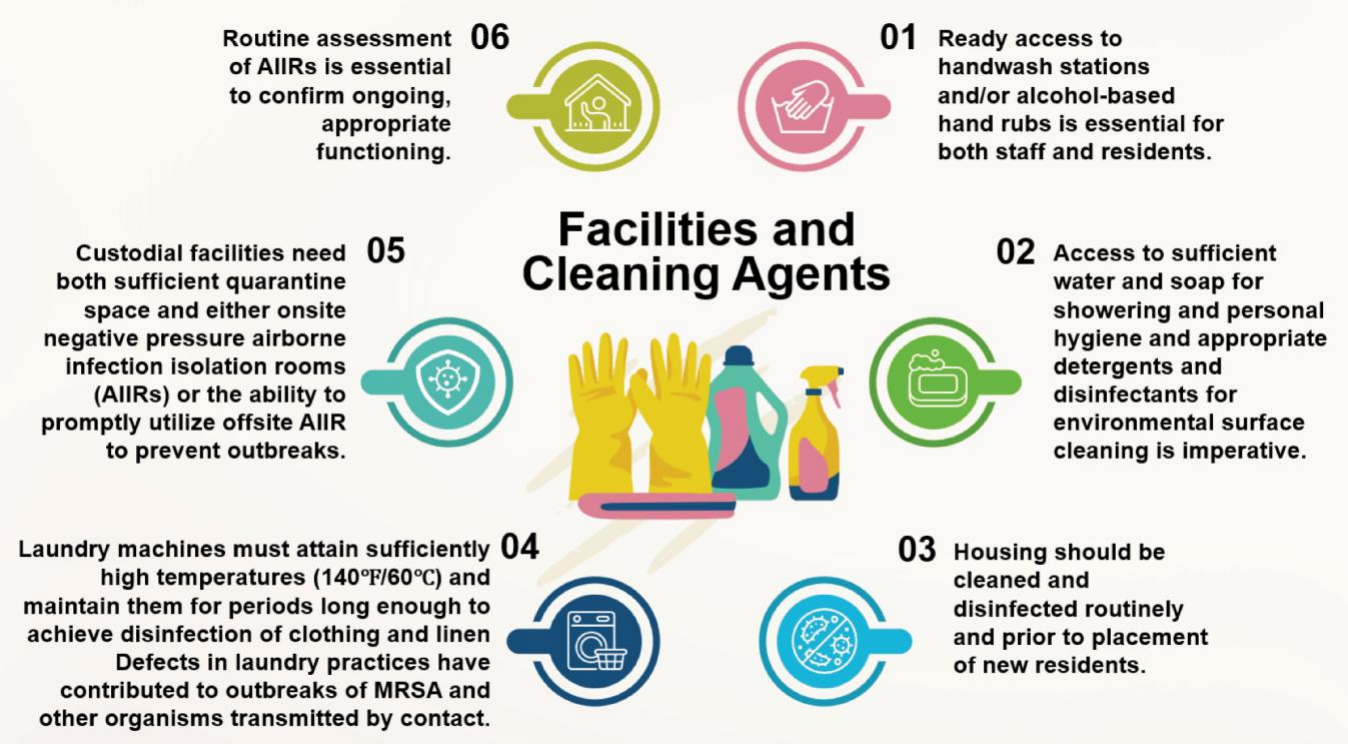
Facilities and cleaning agents.

Trained responsible personnel, comprehensive written policies (Table 3 and Figure 1) and education of staff and residents are critical to the success of an infection control program. Failure to designate staff to monitor and enforce policies contributed to a prison TB outbreak [46]. Training residents to serve as peer educators increases knowledge, reduces risk-inducing behavior, and increases the intention for positive behavior among residents [47–53]. A cluster-randomized trial conducted in Ethiopian prisons [52] demonstrated that involving peer educators in the intervention significantly improved the TB case detection rate.

Box 1. **Definitions and explanations of terms associated with incarceration and detention.**

**Capacity measures:** Three measures of capacity are considered for state and federal prisons. (1) Design capacity—the number of beds the facility can accommodate according to the building designer. (2) Operational capacity—the number of beds the facility can hold as determined by staffing and services. (3) Rated capacity—the number of beds the facility can utilize as designated by an official responsible for rating.

**Jails:** Facilities operated under the jurisdiction of local authorities, including county jails, city jails, and tribal jails under the jurisdiction of tribal authorities. Individuals incarcerated in jails are typically awaiting trial or serving sentences of <1 year. Due to frequent admissions and releases, the annual number of incarcerations occurring in jails greatly exceeds that in prisons.

**Juvenile:** An individual who is under 18 years of age.

**Juvenile residential facilities:** Loosely defined as the different institutions or places where individuals who are accused or convicted of offenses and are under age 18 years may be held for a short or long term.

**Parole:** The practice of releasing convicted individuals who have served a significant portion of their prison sentence to serve out the remainder of their sentence in the community under supervision and in accordance with certain terms or conditions. Violation of the terms of parole is grounds for a return to prison to serve out the remaining term.

**Prisons:** Facilities operated under the jurisdiction of federal or state governments, in which individuals convicted of offences and typically having sentences >1 year are incarcerated. Due to longer stays and less frequent releases, the US prison population is about twice the jail population. (Note that 6 US states run a combined prison–jail system).

**Probation:** A court-ordered period of supervision in the community while under the control, supervision, or care of a correctional agency.

**Resident:** Used in this paper to refer to individuals incarcerated in jails, prisons, and juvenile residential facilities.

*Source: Documents on the Department of Justice's*  https://BJS.gov  *website, including Correctional Populations series.*

**Table 1.** Changes in the United States since 2007 Impacting Infection Control in Carceral Settings

Issue	Comments
Legislation facilitating adoption of information technology innovations	Development of electronic platforms to store data such as patient care registries, dashboards, and health records [9, 10].
	Increased use of telemedicine for specialty care, including infectious disease consultations [11].
Condom availability	Six-fold increase in US state prison residents who have access to condoms [12].
Increased access to MOUD	More than 90% of 23 state prison systems surveyed in 2019 had some form of MOUD in at least 1 prison [13].
Therapeutic advances for TB, HIV, and HCV	HCV pan-genotypic DAAs achieving a cure in over 94% of those treated are now being used in prisons [14].
	HIV integrase strand transfer inhibitors have accelerated viral suppression and resuppression [15].
	HIV preexposure prophylaxis has been initiated in eligible residents awaiting trial [16].
	Briefer regimens for effective treatment of latent TB [17], drug susceptible [18], and multidrug-resistant TB disease [19] now exist.
Infectious disease surveillance	Wastewater-based surveillance has emerged as a useful tool to detect, monitor, and limit outbreaks in carceral facilities [20–27] (Figure 3).
Guidelines for management of infections within carceral settings	The CDC [28] and the Federal Bureau of Prisons [29] have published publicly available documents that serve as resources.
In-prison licensed tattooing services to reduce BBV	Safer tattoo options have been introduced in at least 3 state prison facilities (Missouri, Minnesota, and Delaware) [30–32].

Abbreviations: BBV, blood-borne virus; CDC, Centers for Disease Control and Prevention; DAAs, directly acting antivirals; HCV, hepatitis C virus; HIV, human immunodeficiency virus; MOUD, medication for opioid use disorder; TB, tuberculosis.

**Table 2.** Persisting Infection Control Challenges in US Carceral Settings

Issue	Comments
Inadequate ventilation	Facilitates transmission of respiratory pathogens
Facility design	Facilities are designed primarily to securely hold individuals; infection control is typically not a primary concern in design, resulting in a paucity of isolation and quarantine space.
Overcrowding	In 2022, 16% of jails and 34% of state prison systems and the federal prison system were at or exceeded full capacity, facilitating the transmission of communicable diseases [33, 34].
Burden of chronic health conditions	Up to 50% of residents of carceral facilities enter with health conditions that can increase susceptibility to infections [35].
	Over half have mental illness [36], and up to 25% have cognitive and/or learning disabilities [37], which can impede comprehension and adherence to infection control recommendations.
MOUD availability	MOUD reduces IDU yet remains unavailable in most jails and prisons [13, 38, 39].
	Overdose-related deaths increased more than 450% between 2000 and 2018 in jails [40] and >700% between 2001 and 2018 in prisons [41].
SSPs	SSPs prevent BBV transmission among PWID in prisons [42]; nonetheless, no US prison or jail has implemented them [38, 43].
Screening at entry into jails and prisons	Despite existing recommendations from the CDC [28], few jails screen for infectious diseases at intake [38] or subsequently.
Copayment requirements to access health care	Copayment requirements delay or prevent residents from seeking care [44], increasing the likelihood for infectious disease progression and transmission. Federal prisons and 80% of state prisons still require co-payment for residents accessing care [45]. Some settings waive copay for contagious conditions.
Condom availability	Although California's new condom policy has increased access to condoms, only 2 other state prison systems and 5 US jails reported providing them [12].

Abbreviations: CDC, Centers for Disease Control and Prevention; IDU, injection drug use; MOUD, medication for opioid use disorder; PWID, people who inject drugs; SSPs, Syringe Services Programs.

**Table 3.** Elements of an Effective Infection Prevention Program

Issue	Comments
Training	Provide infection control training to all staff (including custody officers) at time of hire, annually, and whenever there are significant changes
Clinical encounters	Facilitate hand hygiene by providing alcohol hand rubs and hand washing stations with soap and water wherever clinical encounters take place
Multimodal messaging	Utilize a variety of strategies, including posters, brochures, fliers, videos, informational handouts, and face-to-face presentations for staff and residents
Messaging adaptations for special groups and individuals	Culturally sensitive messaging and education contribute to the success of infection control initiatives and interventions [12, 47]. Adapt messaging for those who are illiterate, hearing, or visually impaired and those with cognitive disabilities
	Translate messaging for non-English-speaking residents
Stakeholder engagement	Actively involve staff and residents when developing infection control interventions and solicit feedback for improvements
Inclusion of residents	The use of peer educators with lived experience is more likely to result in behavioral changes [47–53]
Continuous program evaluation	Evaluate infection prevention and control programs at intervals to ensure adherence and effectiveness

## COLLABORATION, COORDINATION, AND OUTBREAK CONTROL

Carceral facilities often lack the expertise necessary to conduct epidemiological surveillance, contact tracing, outbreak management, and notification of reportable diseases. Close working relationships with local, county, and state health departments, public health laboratories, and community hospitals facilitate both prevention and response to outbreaks [54, 55]. During the COVID-19 pandemic, partnerships with public health agencies were crucial in mounting and sustaining the response to COVID-19 in carceral facilities [27, 56–58]. Public health departments also serve to bridge gaps in communication and infection control practices [55] that may exist between city, county, state, and federal facilities, especially during transfers of individuals between jurisdictions. Information technology (IT) applications can facilitate coordination and outbreak control, as the Illinois Department of Public Health demonstrated with a biometric tracking system deployed during the COVID-19 pandemic [27]. The system interacted with electronic medical records (EMRs) to collect health information and enhance infectious disease tracking in jails.

## INFECTIONS TRANSMISSIBLE BY AIRBORNE AND DROPLET ROUTES

Close living quarters and inadequate ventilation create high-risk environments for infectious respiratory illnesses, while the aging prison population and elevated prevalence of comorbidities increase the likelihood of severe complications. (See Table 4 for important aspects of infection prevention and control relevant to influenza and other respiratory illnesses.)

**Table 4.** Key Components of Prevention and Control Programs for Influenza and Other Respiratory Illnesses in Carceral Settings

Subject	Comments
Education	Educate all staff and residents on prevention measures, including hand hygiene, vaccination, and the importance of respiratory hygiene and cough etiquette, with increased emphasis during influenza season
	Encourage the use of face coverings by residents and staff who are coughing or sneezing
Hand hygiene	Provide soap, water, hand-wash stations, and alcohol hand rubs and make them accessible in common and clinical areas
Vaccination	Encourage staff to receive vaccinations for influenza and other respiratory illnesses Offer vaccination to all residents at intake screening and at the beginning of the influenza season, prioritizing the elderly, immunocompromised, and caregivers
Active surveillance and testing for ILI	Conduct surveillance for ILI and use rapid molecular testing for prompt identification of influenza outbreaks Inform local and state health department officials within 24 h of identifying an outbreak
Standard and transmission-based precautions, medical isolation, quarantine, and cohorting	Use standard and droplet precautions during the care of any patient who has suspected or confirmed influenza Identify appropriate locations for isolation and quarantine with established guidelines for their use House suspect and confirmed patients singly may cohort confirmed patients when space is limited
	Implement test-to-program options for suspects to minimize social isolation and improve access to education, employment, and recreation
PPE	Maintain periodic automatic replenishment levels of PPE and other essential supplies to prevent shortages
Ventilation	Assess current ventilation and augment as needed
	Ensure new constructions meet recommended ventilation standards Develop emergency plans for rapid lowering of population density during outbreaks
Restrictions on movement and visiting	Limit or suspend, as necessary, programming, movement into or out of affected units, and visits for or by individuals with respiratory symptoms
Prophylaxis and treatment	Use antiviral agents for treatment and prophylaxis of residents and staff following current guidelines
Access to care	Suspend copay for residents who have communicable diseases
Environment	Increase disinfection of high-touch areas

Abbreviations: ILI, influenza-like illness; PPE, personal protective equipment.

COVID-19 disproportionately impacted carceral settings with an infection rate >5-fold greater in prisons than in the community [59, 60]. COVID-19 cases and death rates were significantly higher among the incarcerated and custodial staff than in the general US population [60, 61]. Carceral systems having strong working relationships with emergency management and public health agencies were more successful in accessing needed resources and adapting to changing guidelines [27]. Robust IT infrastructure facilitated the identification of at-risk patients, immunization strategies, clinical management of exposed and infected patients, and communication with stakeholders. Telehealth enabled clinical encounters despite social distancing and movement restrictions [27, 58]. Increased utilization of alternatives to incarceration and selective decarceration [27, 56, 57, 62] decreased overcrowding, reducing the number of people exposed within carceral facilities. Policies were modified to provide alcohol-based hand sanitizer to residents [63] in monitored or secured wall-mounted dispensers, addressing fire-hazard concerns [55] and the risk of ingestion by residents [64]. Ventilation was improved via installation of high-efficiency particle air filters and ultraviolet germicidal irradiation, decreasing recirculation of air and increasing natural ventilation with outside air [27, 56]. Wastewater-based surveillance for COVID-19 provided a noninvasive method for early detection of COVID-19, facilitating targeted cost-effective interventions [20–27 ] (Figure 3).

Limited space necessitated the use of “Alternative Care Sites” for quarantine and isolation [27] and cohorting individuals with similar infection status. These strategies often reduced residents’ access to visitations, work, school, and recreational activities [57, 65]. For some, isolation resembled punishment and resulted in negative psychological effects [66]. Unintentional negative impacts of infection control strategies and advance communication to residents must be considered [67]. Video visitation and tele-mental health services can mitigate the impact of isolation and quarantine [27, 62, 65, 67, 68].

In 2024, there were 66 confirmed human cases of Highly Pathogenic Avian Influenza (HPAI) A (H5N1) in the United States associated with the ongoing multi-state outbreaks in dairy cattle and poultry [69]. Incarcerated persons work in dairies, egg farms, and meat processing plants. Personal protective equipment (PPE) should be provided to residents who are either in close contact with sick or dead animals or animal feces potentially contaminated with HPAI A (H5N1) or work in farms or regions with confirmed or potentially infected animals [70].

**Figure 2.**
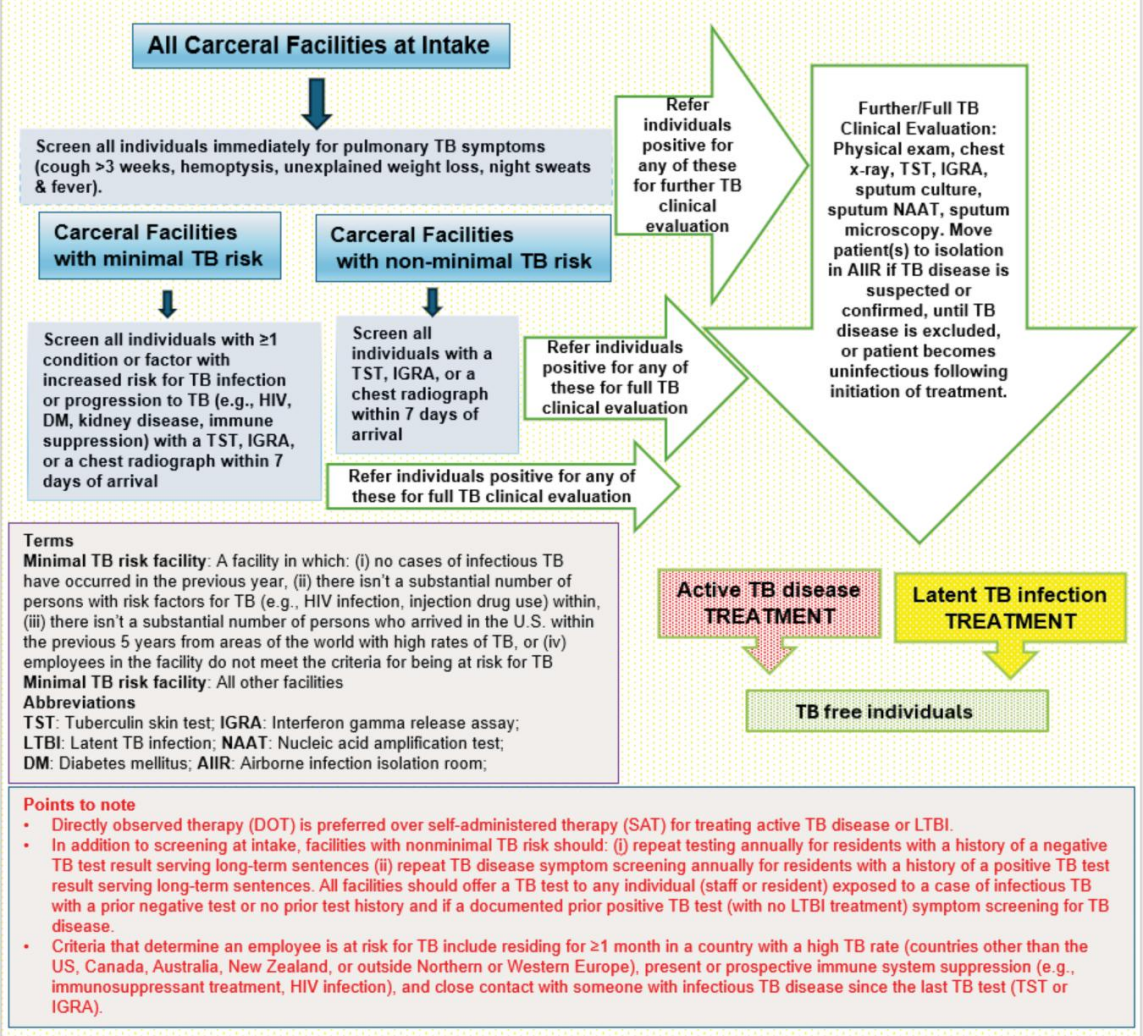
Simplified flow chart for tuberculosis screening at intake in carceral facilities.

With an incidence at least 4 times higher than in the general US population [7], TB persists as a significant problem in carceral settings, resulting in numerous outbreaks [46, 71–74]. Diagnosis is sometimes not made before release [46, 74]. Shared living and sleeping quarters, and the high concentration of individuals with immunocompromising conditions [35] facilitate reactivation and transmission of TB. Foreign born and those who have experienced homelessness are at increased risk for TB disease. Intake screening for active TB disease and latent TB infection (LTBI; Figure 2), and depending on the facility's risk status, annually or when there is an exposure, remains important. Treatment of LTBI is essential to prevent TB disease; the availability of shorter regimens with fewer adverse effects has improved adherence and treatment completion [75]. Deferment of annual TB screening, while focusing on COVID-19 response, contributed to a recent TB outbreak in a state prison system [72]. Improved diagnosis and treatment of HIV has decreased the role of HIV as a precipitant of TB outbreaks in carceral settings [71].

**Figure 3.**
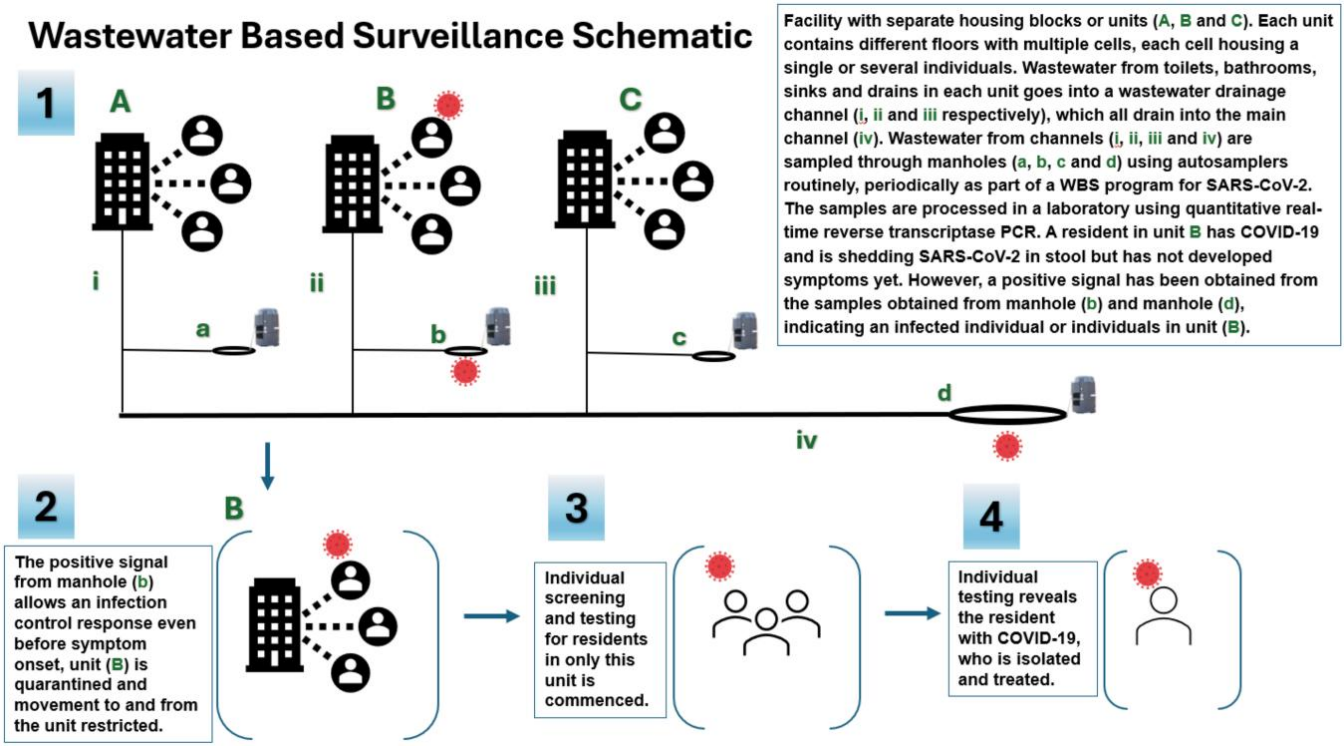
Wastewater-based surveillance (WBS).

## BLOOD-BORNE VIRUSES

HIV and chronic HBV are at least 3 times more prevalent among the incarcerated than in the general US population [76]. HCV viremia is estimated to be 8.7 times more common in state prisons than in the general population [4]. Injection drug use (IDU) [43, 77] and sexual activity [12] routinely occur in jail and prison settings, increasing the likelihood of transmission of blood-borne viruses (BBVs; see Table 5 for screening guidelines). Due to rapid turnover in jail settings, rapid point-of-care opt-out screening for HIV at intake is most effective [5]. Prompt initiation of antiretroviral therapy among newly diagnosed individuals results in rapid viral suppression, making HIV undetectable and untransmissible—essentially, treatment as prevention. In 2019, only 17 AIDS-related deaths were reported from state prisons [78], the lowest since 1991, when data collection began. Preexposure prophylaxis initiation and linkage to care in the community should be offered to at-risk persons in carceral settings in line with Centers for Disease Control and Prevention (CDC) recommendations [79].

**Table 5.** Summarized CDC Guidelines for Opt-Out Screening of Those in Custody

Infectious Agents	Screening Eligibility for Men and Boys	Screening Eligibility for Women and Girls
*N. gonorrhoeae*, *C. trachomatis*	All <30 y	All ≤35 y
*T. vaginalis*	…	All ≤35 y
*T. pallidum*	All, based on local epidemiology	All, based on local epidemiology
HIV (rapid point of care for jails, lab based for prison)	All	
HBV, HCV, *M. tuberculosis*	All	
Mpox	All those with a rash consistent with mpox	
Follow Department of Health and Human Services guidelines when determining frequency: Repeat GC/CT in men up to quarterly Syphilis annually in absence of symptoms Persons with a history of IDU, such as those in MOUD programs: annual HIV, HCV testing [5, 28]		

Abbreviations: CDC, Centers for Disease Control and Prevention; HBV, hepatitis B virus; HCV, hepatitis C virus; HIV, human immunodeficiency virus; IDU, injection drug use; MOUD, medication for opioid use disorder.

Access to directly acting antivirals (DAAs) for the treatment of HCV is limited in many state prisons and jails [14]. DAAs have been used with success in microelimination initiatives in several carceral systems outside the United States [47, 80]. The California prison system has provided opt-out HCV testing for all new arrivals since 2016. Between July 2018 and July 2023, >28 000 patients were treated with DAAs, >90% of these were cured, and HCV prevalence among the incarcerated declined from 14% to 4.1% [81]. Those at highest risk for HCV infections are also more likely to encounter the criminal legal system [47] and may rotate between the community and jails. Including jails in “test and treat” programs can reduce the HCV burden in the community. Populations at high risk of HIV and viral hepatitis commonly have reduced access to healthcare in the community [82], making their jail stay an opportunity to initiate diagnosis, treatment, and prevention.

Offering the HBV vaccine to nonimmune individuals and treating chronically infected persons is consistent with current American Association for the Study of Liver Disease guidelines. Harm reduction programs reduce transmission of BBVs among people who inject drugs (PWIDs) in carceral settings [38, 42, 43]. Syringe services programs (SSPs) [38, 43] and medication for opioid use disorder (MOUD) can prevent BBV among incarcerated PWID [38]. Condoms prevent sexually transmitted infections (STIs), including BBV, and are safe and cost-effective [12]. Exposure to blood and other potentially contagious body fluids is common in carceral settings. Prompt evaluation of exposed staff and residents and timely initiation of HIV and HBV postexposure prophylaxis are essential [1].

## OTHER SEXUALLY TRANSMITTED INFECTIONS

Sexually transmitted infections are more common among persons entering and residing in jails, prisons, and juvenile facilities [5, 28, 83] than in the general population (see Table 5 for screening guidelines). Screening identifies those infected, facilitates treatment, and prevents transmission. Routine opt-out testing results in higher rates of screening among at-risk populations [5]. Jail-based STI screen-and-treat programs have the potential to reduce STI prevalence in associated external communities [79, 84].

## VACCINES AND POSTEXPOSURE PROPHYLAXIS

Carceral settings should offer nonimmune residents immunizations for HBV, hepatitis A virus (HAV), human papillomavirus (HPV), measles, varicella, mumps, influenza, meningitis, and COVID-19 upon intake and seasonally when indicated. Nonimmune staff should be encouraged to receive vaccinations from their healthcare providers. In juvenile detention settings, costs are fully covered by the vaccines for children program for adolescents under 18 years of age [5], while vaccines for incarcerated adults may be purchased using funds from the Federal Section 317 Immunization Program [85]. Although COVID-19 vaccination was prioritized [86, 87] for those living and working in carceral settings, low uptake was generally reported among residents and custody staff, with distrust being a major factor [88, 89]. Vaccine mandates have been explored in carceral settings to increase uptake among staff who often have vaccination rates lower than residents [88–90], resulting in legal challenges and staff resignations [89, 90], and depleting an already insufficient workforce. Efforts at vaccine education and counseling for those who work and live in carceral settings using thought leaders and influencers may improve uptake [88–90].

## SKIN AND SOFT TISSUE INFECTIONS AND INFESTATIONS

Overcrowding; restricted access to soap, showers, and cleaning supplies; poor personal hygiene; sharing of personal items such as razors and tattoo equipment; deficient environmental cleaning; and defective laundry processes can all contribute to the occurrence of skin and soft tissue infections (SSTIs) and outbreaks [91]. Copayments can delay prompt wound care, thereby contributing to the progression of infections and infectious disease outbreaks [44].

Dermatophyte skin infections are the most common dermatologic infection in some carceral settings [92–94] and vary according to the degree of overcrowding and the lack of soap, water, and other items for personal and environmental hygiene. [93]. Methicillin-resistant *Staphylococcus aureus* (MRSA) is an important cause of SSTIs in carceral settings [95, 96]. MRSA SSTIs have been misidentified as spider bites [1]. Intake screening for SSTIs facilitates prompt treatment, decreasing the likelihood of transmission.

IDU is a significant risk factor for SSTI, infective endocarditis, osteomyelitis, and bacterial CNS infections [97]. Improved access to MOUD decreases IDU-related SSTI. Routine harm reduction education should include hand hygiene, cleaning skin prior to injection, and not using any equipment contaminated by reuse, saliva, soil, or water [98]. In California, SSPs, incorporating substance use treatment referrals, HIV and hepatitis testing, overdose prevention education, and safe disposal of used syringes, have significantly contributed to reducing the transmission of HIV, HBV, and HCV and preventing SSTI among PWID [99]. SSP participants are 5 times more likely to enter drug treatment and 3 times more likely to reduce or stop injecting than those who have never accessed an SSP [100, 101]. Carceral settings should consider implementing SSP as a component of infection control and prevention programs.

Ectoparasite infestations with *Sarcoptes scabiei* and *Pediculus humanus* frequently occur in carceral settings. Reduced vigilance for scabies and human lice may result in severe cases and outbreaks [102, 103]. Essential components of an effective ectoparasite control program include intake screening, contact precautions, prompt treatment and contact investigations, and decontamination of clothing, bedding, and other personal items [29].

## *CLOSTRIDIOIDES DIFFICILE* AND MULTIDRUG-RESISTANT ORGANISMS

*Clostridioides difficile* and multidrug-resistant organisms (MDROs) are a serious concern in carceral settings due to crowding and shared resources, leading to increased risks of infection transmission. Suspected or confirmed infections with *C. difficile* and infections with targeted MDROs, such as *Candida auris* and carbapenemase-producing organisms, should trigger containment measures, including isolation and treatment of infected persons, contact precautions, and enhanced environmental cleaning and disinfection with appropriate agents. CDC guidelines for the containment of MDROs [104, 105] and *C. difficile* [106] should be adapted to the carceral facility setting. If transfer of infected individuals must take place, appropriate precautions should be put in place and advance notification provided to the receiving facility and the jurisdictional public health departments [104].

## ANTIMICROBIAL STEWARDSHIP

Increasing cases of infections due to MDROs in carceral settings [104] highlight the need for engaging in antimicrobial stewardship principles and practices to optimize the use of antibiotics, reduce the emergence of drug-resistant organisms, and improve patient outcomes. A review of the Bureau of Prisons (BOP) antimicrobial stewardship program [107], evaluating its impact over 6 years, found a 25% reduction in antibiotic prescriptions. Helpful CDC resources for setting up antimicrobial stewardship programs in healthcare settings [108] and BOP resources specifically for carceral settings [29] are available.

See Table 6 for key components of an antimicrobial stewardship program in carceral settings.

**Table 6.** Key Components of an Antimicrobial Stewardship Program in Carceral Settings

Subject	Comments
Antimicrobial use	Implement antimicrobial prescribing guidelines. Prescribe antimicrobials only when necessary and with the appropriate drug, dosage, and shortest duration required Track antimicrobial consumption, resistance patterns, and outcomes to identify areas for improvement
	Encourage clinicians to de-escalate antimicrobial therapy when possible, moving from broad-spectrum to narrow-spectrum antimicrobials once the infection is identified
Education	Educate staff and residents on the importance of antibiotics/antimicrobials, resistance, and proper use
Diagnostics	Implement better diagnostic tools to accurately identify the cause of infections, allowing for more targeted antimicrobial therapy
Collaboration	Foster collaboration between carceral clinicians, residents, and local hospitals to promote a culture of appropriate antimicrobial stewardship

## FOODBORNE DISEASES AND GASTROINTESTINAL ILLNESSES

The rate of foodborne disease outbreaks and the number of illnesses per outbreak are >5-fold greater in carceral settings than in the general US population [109]. Most culinary and scullery work within carceral settings is performed by residents, many of whom receive little if any training. Deficiencies in food safety and handling may result in the introduction of foodborne pathogens. Such deficiencies were implicated in reported *Salmonella* outbreaks involving mechanically separated chicken in multiple carceral facilities [110–112]. One report demonstrated that cooking temperatures were not routinely monitored, and raw food was still frozen when cooking commenced [112]. One in 7 outbreaks of foodborne illness in carceral facilities implicated food handlers as the source of contamination [109]. See Table 7 for specific issues associated with foodborne illness in carceral settings. Infection control programs should educate residents regarding the causes of foodborne illness and best practices for food handling. The CDC provides a useful document to guide food safety in carceral facilities [113].

**Table 7.** Notable Causes of Foodborne Illness in Carceral Settings

Issue	Comments
Improper storage by residents	Perishable food is commonly stored by residents for extended hours despite the lack of refrigeration in housing units
Food preparation and handling	Incomplete thawing of frozen food prior to cooking Failure to monitor temperatures while preparing food to ensure adequate cooking temperatures were reached Leaving food at room temperature for multiple hours Insufficient cleaning of instruments Insufficient dishwashing temperatures Inadequate hand hygiene
Lack of access to disinfectants	Norovirus outbreaks are common in carceral settings and often spread rapidly Although sodium hypochlorite (bleach) is recommended for disinfection of high-touch surfaces and contaminated areas [114], the use of bleach is often restricted in carceral settings [1], and written policies may be required to ensure ready access
Unauthorized alcohol production and consumption	Unauthorized production of prison-brewed alcohol, (“pruno” or “hooch”), is common in carceral settings. Consumption has resulted in botulism with severe morbidity and significant healthcare costs [115–117]. Education and altering or eliminating the materials used in “pruno” production have been attempted as a control measure [115]

## WASTEWATER-BASED SURVEILLANCE

During the COVID-19 pandemic, facility-based WBS (Figure 3) was a useful infection control tool within carceral settings [20–27]. WBS can serve as an early warning system, allowing an infection control response that precedes the onset of symptoms of respiratory infection, thereby reducing transmission and curtailing outbreaks [21]. WBS facilitates a focused, streamlined response, saving resources and reducing the number of residents impacted by quarantine or isolation [21].

## CONCLUSIONS

The past 20 years have witnessed significant advances in the prevention, diagnosis, and treatment of common communicable diseases in carceral settings. Decreasing incarceration rates, improved diagnostics and therapeutics, adoption of EMRs, and improved implementation, education, and adherence to infection prevention and control policies and procedures have resulted in public health improvements for those working and residing in custodial facilities.

Eradication of HCV is achievable as is a continued decline in HIV-associated morbidity and mortality. Improvement in vaccination rates and in the prevention, diagnosis, and treatment of infectious diseases will benefit both the incarcerated and the communities to which they return. Increased access to MOUD and harm reduction strategies is an essential component of infection control in jails and prisons. The COVID-19 pandemic demonstrated that carceral settings remain vulnerable to outbreaks, especially involving airborne pathogens, and that there is a bidirectional impact of health between carceral settings and outside communities. Collaboration with outside public health experts will foster mutual understanding of optimal public health strategies in carceral settings.

## Supplementary Material

ciaf479_Supplementary_Data

## Supplementary Data

Supplementary materials are available at *Clinical Infectious Diseases* online. Consisting of data provided by the authors to benefit the reader, the posted materials are not copyedited and are the sole responsibility of the authors, so questions or comments should be addressed to the corresponding author.
