# Supplementary material for: Infection Control in Carceral Facilities
Source: Clin Infect Dis. 2025 Oct 21;82(4):e781–90. doi: 10.1093/cid/ciaf479 (PMC13131935; doi:10.1093/cid/ciaf479)
Supplement: ciaf479_Supplementary_Data [file ciaf479_supplementary_data.docx]

**Supplementary document with references 41-117 for Infection Control in Carceral Settings**

41. Carson EA. Mortality in State and Federal Prisons, 2001–2018 - Statistical Tables. U.S. Department of Justice, Office of Justice Programs, Bureau of Justice Statistics, 2021: 34.

42. Lazarus JV, Safreed-Harmon K, Hetherington KL, et al. Health Outcomes for Clients of Needle and Syringe Programs in Prisons. Epidemiol Rev **2018**; 40:96–104.

43. Armstrong-Mensah E, Dada D, Rupasinghe R, Whately H. Injecting substance use in prisons in the United States: a case for needle exchange programs. Am J Drug Alcohol Abuse **2021**; 47:273–279.

44. Lupez EL, Woolhandler S, Himmelstein DU, et al. Health, Access to Care, and Financial Barriers to Care Among People Incarcerated in US Prisons. JAMA Intern Med **2024**;

45. Prison Policy Initiative. COVID looks like it may stay. That means prison medical copays must go. 2022. Available at: https://www.prisonpolicy.org/blog/2022/02/01/pandemic_copays/. Accessed 4 March 2024.

46. Lambert LA, Espinoza L, Haddad MB, et al. Transmission of Mycobacterium tuberculosis in a Tennessee Prison, 2002-2004. Journal of Correctional Health Care **2008**; 14:39–47.

47. Akiyama MJ, Kronfli N, Cabezas J, et al. Hepatitis C elimination among people incarcerated in prisons: challenges and recommendations for action within a health systems framework. Lancet Gastroenterol Hepatol **2021**; 6:391–400.

48. Thornton K, Sedillo ML, Kalishman S, Page K, Arora S. The New Mexico Peer Education Project: Filling a Critical Gap in HCV Prison Education. Journal of Health Care for the Poor and Underserved **2018**; 29:1544–1557.

49. Janota AD, Hibbard PF, Meadows ME, et al. Peer Education as a Tool to Improve Health Knowledge for People Who Are Incarcerated: A Secondary Analysis of Data From the Indiana Peer Education Program ECHO. Journal of Correctional Health Care **2024**; 30:226–237.

50. Stasi C, Monnini M, Cellesi V, et al. Ways to promote screening for hepatitis B virus and accelerated vaccination schedule in prison: Training, information, peer education. Rev Epidemiol Sante Publique **2022**; 70:25–30.

51. Bagnall A-M, South J, Hulme C, et al. A systematic review of the effectiveness and cost-effectiveness of peer education and peer support in prisons. BMC Public Health **2015**; 15:290.

52. Adane K, Spigt M, Winkens B, Dinant G-J. Tuberculosis case detection by trained inmate peer educators in a resource-limited prison setting in Ethiopia: a cluster-randomised trial. The Lancet Global Health **2019**; 7:e482–e491.

53. Wright N, Bleakley A, Butt C, et al. Peer health promotion in prisons: a systematic review. International Journal of Prisoner Health **2011**; 7:37–51.

54. Beaudry G, Zhong S, Whiting D, Javid B, Frater J, Fazel S. Managing outbreaks of highly contagious diseases in prisons: a systematic review. BMJ Glob Health **2020**; 5:e003201.

55. Kendig NE, Bur S, Zaslavsky J. Infection Prevention and Control in Correctional Settings - Volume 30, Supplement—March 2024 - Emerging Infectious Diseases journal - CDC. Available at: https://wwwnc.cdc.gov/eid/article/30/13/23-0705_article. Accessed 6 May 2025.

56. Barnert E, Kwan A, Williams B. Ten Urgent Priorities Based on Lessons Learned From More Than a Half Million Known COVID-19 Cases in US Prisons. Am J Public Health **2021**; 111:1099–1105.

57. Novisky MA, Tostlebe J, Pyrooz D, Sanchez JA. “The COVID-19 pandemic and operational challenges, impacts, and lessons learned: a multi-methods study of U.S. prison systems”. Health & Justice **2023**; 11:51.

58. Waddell C, Meehan A, Schoonveld M, et al. Lessons Learned from COVID-19 Response in Correctional and Detention Facilities. Emerg Infect Dis **2024**; 30:S5–S12.

59. Wang EA, Zenilman J, Brinkley-Rubinstein L. Ethical Considerations for COVID-19 Vaccine Trials in Correctional Facilities. JAMA **2020**; 324:1031–1032.

60. Saloner B, Parish K, Ward JA, DiLaura G, Dolovich S. COVID-19 Cases and Deaths in Federal and State Prisons. JAMA **2020**; 324:602–603.

61. National Academies of Sciences, Engineering, and Medicine. Decarcerating Correctional Facilities during COVID-19: Advancing Health, Equity, and Safety. Washington (DC): National Academies Press (US), 2020. Available at: http://www.ncbi.nlm.nih.gov/books/NBK566319/. Accessed 20 October 2024.

62. Novisky MA, Narvey CS, Semenza DC. Institutional Responses to the COVID-19 Pandemic in American Prisons. Victims & Offenders **2020**; 15:1244–1261.

63. Federal Bureau of Prisons. COVID-19 Pandemic Response Plan. 2021; Available at: https://www.bop.gov/foia/docs/COVID_pandemic_plan_docs_v6_2021_07_16.pdf. Accessed 19 April 2025.

64. Doyon S, Welsh C. Intoxication of a Prison Inmate with an Ethyl Alcohol–Based Hand Sanitizer. New England Journal of Medicine **2007**; 356:529–530.

65. Johnson L, Gutridge K, Parkes J, Roy A, Plugge E. Scoping review of mental health in prisons through the COVID-19 pandemic. BMJ Open **2021**; 11:e046547.

66. Rosenberg A, Puglisi LB, Thomas KA, et al. “It’s just us sitting there for 23 hours like we done something wrong”: Isolation, incarceration, and the COVID-19 pandemic. PLoS One **2024**; 19:e0297518.

67. Shiple C, Eamranond PP. Letter to the Editor—The disproportionate negative impacts of COVID‐19 on the mental health of prisoners. J Forensic Sci **2021**; 66:413–414.

68. Hewson T, Shepherd A, Hard J, Shaw J. Effects of the COVID-19 pandemic on the mental health of prisoners. The Lancet Psychiatry **2020**; 7:568–570.

69. Centers for Disease Control and Prevention. H5 Bird Flu: Current Situation. 2024. Available at: https://www.cdc.gov/bird-flu/situation-summary/index.html. Accessed 2 January 2025.

70. Centers for Disease Control and Prevention. Interim Guidance for Employers to Reduce Exposure to Novel Influenza A (Such as H5N1 Bird Flu) for People Working with or Exposed to Animals. 2024. Available at: https://www.cdc.gov/bird-flu/prevention/worker-protection-ppe.html. Accessed 2 January 2025.

71. Stewart RJ, Raz KM, Burns SP, et al. Tuberculosis Outbreaks in State Prisons, United States, 2011–2019. Am J Public Health **2022**; 112:1170–1179.

72. Stalter RM, Pecha M, Dov L, et al. Tuberculosis Outbreak in a State Prison System - Washington, 2021-2022. MMWR Morb Mortal Wkly Rep **2023**; 72:309–312.

73. Séraphin MN, Didelot X, Nolan DJ, et al. Genomic Investigation of a Mycobacterium tuberculosis Outbreak Involving Prison and Community Cases in Florida, United States. Am J Trop Med Hyg **2018**; 99:867–874.

74. Sosa LE, Lobato MN, Condren T, Williams MN, Hadler JL. Outbreak of tuberculosis in a correctional facility: consequences of missed opportunities. Int J Tuberc Lung Dis **2008**; 12:689–691.

75. Wheeler C, Mohle-Boetani J. Completion Rates, Adverse Effects, and Costs of a 3-Month and 9-Month Treatment Regimen for Latent Tuberculosis Infection in California Inmates, 2011-2014. Public Health Rep **2019**; 134:71s–79s.

76. Centers for Disease Control and Prevention (CDC). Public Health Considerations for Correctional Health. 2024. Available at: https://www.cdc.gov/correctional-health/about/index.html. Accessed 21 October 2024.

77. Rowell-Cunsolo TL, Sampong SA, Befus M, Mukherjee DV, Larson EL. Predictors of Illicit Drug Use Among Prisoners. Subst Use Misuse **2016**; 51:261–267.

78. Maruschak LM. HIV in Prisons, 2020 – Statistical Tables. U.S. Department of Justice, Office of Justice Programs, Bureau of Justice Statistics, 2022: 33. Available at: https://bjs.ojp.gov/content/pub/pdf/hivp20st.pdf. Accessed 20 October 2024.

79. Workowski KA, Bachmann LH, Chan PA, et al. Sexually Transmitted Infections Treatment Guidelines, 2021. MMWR Recomm Rep **2021**; 70:1–187.

80. Mongale E, Allen S, Brew I, et al. Development and optimisation of a reception testing protocol designed to eliminate HCV in the UK prison population. JHEP Reports **2024**; 6:100937.

81. Lucas KD, Krawiec A, Wada J, Kanan RJ. The hepatitis C care cascade in California state prisons: Screening and treatment scale-up and progress toward elimination, 2016–2023. Clinical Liver Disease **2024**; 23:e0117.

82. Nijhawan AE. Infectious Diseases and the Criminal Justice System: A Public Health Perspective. The American journal of the medical sciences **2016**; 352:399.

83. Wolf C, Clifton J, Sheng X. Screening for Chlamydia and Gonorrhea in Youth Correctional Facilities, Utah, USA. Emerging Infectious Diseases **2024**; 30:S62.

84. Barry PM, Kent CK, Scott KC, Goldenson J, Klausner JD. Is jail screening associated with a decrease in Chlamydia positivity among females seeking health services at community clinics?-San francisco, 1997-2004. Sex Transm Dis **2009**; 36:S22-28.

85. Granade CJ, Crawford NE, Banks M, Graitcer S. Analysis of the Federal Section 317 Immunization Program and Routine Adult Immunization Activities, United States, 2022-2023. Public Health Rep **2024**; 139:626–634.

86. Jain V, Schwarz L, Lorgelly P. A Rapid Review of COVID-19 Vaccine Prioritization in the U.S.: Alignment between Federal Guidance and State Practice. Int J Environ Res Public Health **2021**; 18:3483.

87. National Academies of Sciences, Engineering, and Medicine. Framework for Equitable Allocation of COVID-19 Vaccine. Washington, DC: The National Academies Press, 2020. Available at: https://nap.nationalacademies.org/catalog/25917/framework-for-equitable-allocation-of-covid-19-vaccine. Accessed 20 May 2025.

88. Kramer C, Song M, Sufrin CB, Eber GB, Rubenstein LS, Saloner B. COVID-19 vaccination hesitancy and uptake: Perspectives from people released from the Federal Bureau of Prisons. Vaccine **2023**; 41:1408–1417.

89. Kraus C, Guardado R, Wurcel AG. Corrections Officers’ and Sheriffs’ Perceptions of COVID-19 Vaccine Operationalization. J Correct Health Care **2023**; 29:150–155.

90. Spaulding AC, Zawitz C. Vaccination in Prisons and Jails: Corrections Needed in Future Plans. Clinical Infectious Diseases **2022**; 75:e846–e848.

91. Elias AF, Chaussee MS, McDowell EJ, Huntington MK. Community-based intervention to manage an outbreak of MRSA skin infections in a county jail. J Correct Health Care **2010**; 16:205–15.

92. Coury C, Kelly B. Prison dermatology: experience in the Texas Department of Criminal Justice dermatology clinic. J Correct Health Care **2012**; 18:302–308.

93. Ferris M, Islam RK, Huynh TN, et al. An international review of skin conditions in incarcerated persons. JAAD Reviews **2024**; 2:113–126.

94. Oninla OA, Onayemi O. Skin infections and infestations in prison inmates. Int J Dermatol **2012**; 51:178–81.

95. David MZ, Siegel JD, Henderson J, et al. A randomized, controlled trial of chlorhexidine-soaked cloths to reduce methicillin-resistant and methicillin-susceptible Staphylococcus aureus carriage prevalence in an urban jail. Infect Control Hosp Epidemiol **2014**; 35:1466–73.

96. Popovich KJ, Thiede SN, Zawitz C, et al. Genomic Epidemiology of MRSA During Incarceration at a Large Inner-City Jail. Clin Infect Dis **2021**; 73:e3708–e3717.

97. McCarthy NL, Baggs J, See I, et al. Bacterial Infections Associated With Substance Use Disorders, Large Cohort of United States Hospitals, 2012–2017. Clinical infectious diseases : an official publication of the Infectious Diseases Society of America **2020**; 71:e37.

98. Hartnett KP, Jackson KA, Felsen C, et al. Bacterial and Fungal Infections in Persons Who Inject Drugs — Western New York, 2017. Morbidity and Mortality Weekly Report **2019**; 68:583.

99. California Department of Public Health, Center for Infectious Diseases, Office of AIDS. Factsheet - Syringe Services Programs in California: An Overview. 2022; Available at: https://www.cdph.ca.gov/Programs/CID/DOA/CDPH%20Document%20Library/SSP_Factsheet.pdf. Accessed 21 October 2024.

100. Hagan H, McGough JP, Thiede H, Hopkins S, Duchin J, Alexander ER. Reduced injection frequency and increased entry and retention in drug treatment associated with needle-exchange participation in Seattle drug injectors. Journal of Substance Abuse Treatment **2000**; 19:247–252.

101. Strathdee SA, Celentano DD, Shah N, et al. Needle-exchange attendance and health care utilization promote entry into detoxification. Journal of Urban Health : Bulletin of the New York Academy of Medicine **1999**; 76:448.

102. Lawsuit: Tennessee jail infested by scabies. 2017. Available at: https://apnews.com/general-news-13873ad4365c44179d85d3a8e4135640. Accessed 2 May 2025.

103. US Justice Department Finds Rights Violated at Atlanta Jail. Available at: //www.usnews.com/news/top-news/articles/2024-11-14/us-justice-department-finds-rights-violated-at-atlanta-jail. Accessed 2 May 2025.

104. Hennessee I, Forsberg K, Erskine J, et al. Candida auris in US Correctional Facilities - Volume 30, Supplement—March 2024 - Emerging Infectious Diseases journal - CDC. Available at: https://wwwnc.cdc.gov/eid/article/30/13/23-0860_article. Accessed 2 May 2025.

105. Centers for Disease Control and Prevention. Containment strategy: interim guidance for a public health response to contain novel or targeted multidrug-resistant organisms (MDROs). 2022; Available at: https://www.cdc.gov/healthcare-associated-infections/media/pdfs/Health-Response-Contain-MDRO-H.pdf. Accessed 2 May 2025.

106. Centers for Disease Control and Prevention. Clinical Guidance for C. diff Prevention in Acute Care Facilities. 2024. Available at: https://www.cdc.gov/c-diff/hcp/clinical-guidance/index.html. Accessed 5 May 2025.

107. Long MJ, LaPlant BN, McCormick JC. Antimicrobial stewardship in the Federal Bureau of Prisons: Approaches from the national and local levels. Journal of the American Pharmacists Association **2017**; 57:241–247.

108. CDC. Core Elements of Antibiotic Stewardship. 2024. Available at: https://www.cdc.gov/antibiotic-use/hcp/core-elements/index.html. Accessed 23 April 2025.

109. Marlow MA, Luna-Gierke RE, Griffin PM, Vieira AR. Foodborne Disease Outbreaks in Correctional Institutions-United States, 1998-2014. Am J Public Health **2017**; 107:1150–1156.

110. Taylor AL, Murphree R, Ingram LA, et al. Multidrug-Resistant *Salmonella* Heidelberg Associated with Mechanically Separated Chicken at a Correctional Facility. Foodborne Pathogens and Disease **2015**; 12:950–952.

111. Hutchinson JA, Wheeler C, Mohle-Boetani JC. Outbreak epidemiologically linked with a composite product of beef, mechanically separated chicken and textured vegetable protein contaminated with multiple serotypes of Salmonella enterica including multidrug-resistant Infantis, California 2016. Epidemiol Infect **2018**; 146:430–436.

112. Oppegard SJ, Bethke AR, Davy BA, Johnson AE, Daniel JL, Holmes SE. Notes from the Field: Outbreak of Salmonella Enteritidis at a Correctional Facility Using Mechanically Separated Chicken — Nebraska, 2022. MMWR Morb Mortal Wkly Rep **2022**; 71:908–909.

113. Centers for Disease Control and Prevention. Model Food Safety Practices for Correctional Facilities. 2024; Available at: https://www.cdc.gov/correctional-health/media/pdfs/2025/02/Model-Food-Safety-Practices-for-Correctional-Facilities_508.pdf. Accessed 14 May 2025.

114. Barclay L, Park GW, Vega E, et al. Infection control for norovirus. Clin Microbiol Infect **2014**; 20:731–740.

115. Yasmin S, Adams L, Briggs G, et al. Outbreak of Botulism After Consumption of Illicit Prison-Brewed Alcohol in a Maximum Security Prison—Arizona, 2012. J Correct Health Care **2015**; 21:327–334.

116. Rao AK, Walters M, Hall J, et al. Outbreak of Botulism Due to Illicit Prison-Brewed Alcohol: Public Health Response to a Serious and Recurrent Problem. Clinical Infectious Diseases **2018**; 66:S85–S91.

117. McCrickard L. Notes from the Field: Botulism Outbreak from Drinking Prison-Made Illicit Alcohol in a Federal Correctional Facility — Mississippi, June 2016. MMWR Morb Mortal Wkly Rep **2017**; 65. Available at: https://www.cdc.gov/mmwr/volumes/65/wr/mm6552a8.htm. Accessed 13 September 2023.
